# Impact of estimated glomerular filtration rate on long-term clinical outcomes among Chinese patients with atrial fibrillation

**DOI:** 10.1186/s12872-020-01786-6

**Published:** 2020-11-19

**Authors:** Huaibin Wan, Juan Wang, Yanmin Yang, Xin Fan, Dongdong Chen, Ning Bian

**Affiliations:** 1 Department of Cardiology, Dongguan People’s Hospital, Southern Medical University, Guangdong, China; 2 Emergency and Intensive Care Center, Fuwai Hospital, National Center for Cardiovascular Diseases, Chinese Academy of Medical Sciences and Peking Union Medical College, Beijing, China; 3 Department of Cardiology, First Affiliated Hospital, Jinan University, Guangdong, China

**Keywords:** Estimated glomerular filtration rate, Atrial fibrillation, Survival

## Abstract

**Background:**

Estimated glomerular filtration rate (eGFR) is a widely accepted indicator of renal function. The aim of this study was to evaluate the relationship between eGFR and 3-year clinical outcomes among Chinese patients with atrial fibrillation (AF).

**Methods:**

We retrospectively studied 433 consecutive Chinese patients with AF (51.0% males, mean age 65.6 ± 13.2 years) between February 2013 and December 2017. Baseline clinical data were collected according to medical records. eGFR was calculated by MDRD equation for Chinese patients according to baseline age, sex and serum creatinine. The primary clinical outcome of interest was all-cause mortality.

**Results:**

During a median follow-up period of 3.1 (0.5–4.5) years, 73 deaths (16.9%) were recorded. Multivariate Cox regression analyses indicated that eGFR was independently associated with all-cause death in total population [hazard ratio (HR) 0.984; 95% confidence interval (CI) 0.972–0.995, P = 0.006] and patients free of valvular heart diseases (VHDs) (HR 0.975; 95% CI 0.959–0.992, P = 0.003), but not with VHDs. A receiver operating characteristic (ROC) analysis revealed that reduced eGFR predicted all-cause mortality with areas under the ROC curve of 0.637 (95% CI 0.539–0.735, P = 0.004) in AF patients free of VHDs.

**Conclusions:**

eGFR is an independent predictor of 3-year all-cause mortality among Chinese patients with AF, especially among those patients free of VHDs.

## Background

Atrial fibrillation (AF) is the most common sustained arrhythmia with increased risks of stroke and systemic embolism, accounting for about one third of all hospitalized adults with arrhythmias [1, 2]. The prevalence of AF is increasing with age and various cardiovascular risk factors [1, 2]. Glomerular filtration rate (GFR), which can be estimated from age, sex and serum creatinine (estimated GFR, eGFR), is a widely accepted indicator of renal function [3]. It has been identified that eGFR was independently as well as jointly with other indicators providing statistically significant improvement in predicting cardiovascular events [4–6]. Previous studies have shown that renal impairment and reduced eGFR were risk factors for developing AF [7, 8]. In patients after AF catheter ablation, there was an inverse association between eGFR levels and AF recurrences [9]. And eliminating AF by catheter ablation was beneficial to renal function improving over a 5-year follow-up study [10].

AF and chronic kidney disease (CKD) frequently coexist [11]. Therefore, concurrent management of AF and CKD should be required for preventing adverse cardiovascular events. In the EurObservational Research Programme AF General Pilot Registry (EORP-AF), even mild or moderate renal impairments were associated with an increased risks of cardiovascular events and death [12]. However, no investigation regarding the relationship between renal impairment and long-term clinical outcomes has been conducted in general Chinese patients with AF. Therefore, in this study, we evaluated the impact of eGFR on long-term mortality in Chinese patients with AF.

## Methods

### Study population and design

This was a single center cohort study designed to evaluate the long-term prognostic value of eGFR among consecutive Chinese patients with AF. The patient data with AF were retrieved and obtained anonymously from the hospital medical record datasets of the First affiliated hospital of Jinan University between February 2013 and December 2017. The inclusion criteria were as follows: ≥ 18 years old, at least one 12-lead electrocardiogram indicated AF, had comprehensive medical records and underwent a systemic physical examination, baseline laboratory tests and transthoracic echocardiography. The exclusion criteria were as follows: congenital heart diseases, hepatic insufficiency (serum alanine aminotransferase (ALT) or total bilirubin levels > 1.5 times the upper limit of normal), combined with acute or chronic infectious diseases, blood disorders, requiring iron supplementation, malignant illness, thyroid or mental disorders. The identifiable personal privacy and contact information were hidden when exported from the hospital medical record datasets. This study is exempt from the need for informed consent according to China's "Ethical Review Approaches for Biomedical Research Involving Humans" 2016, Article 39(1) [13]. The study was approved by the ethics committee of the First affiliated hospital of Jinan University and was conducted in accordance with the Declaration of Helsinki.

### Data collection

The demographic and baseline clinical characteristics, including age, gender, smoking and drinking statuses, histories of hypertension, diabetes mellitus, hypercholesterolemia, coronary diseases, heart failure, stroke, types of AF, blood pressure, heart rate, body mass index (calculated via the formula: BMI = weight (kg)/[height (m)]^2^) were recorded. AF described in terms of the duration of episodes and using a simplified scheme as follows: paroxysmal AF indicates that an episode of AF is self-terminating or cardioverted within 7 days; persistent AF, sustained longer than 7 days and permanent AF, continuous AF lasts longer than 1 year regardless of the therapeutic attitudes of individual patient and physician [1, 2]. Based on a functional EHRA (Evaluated Heart valves, Rheumatic or Artificial) categorization, patients with rheumatic moderate–severe mitral stenosis or mechanical prosthetic valve replacement were defined as EHRA Type 1 valvular heart diseases (VHDs) and EHRA Type 2 refers to the other individuals with VHDs. [1, 14]. For patients with EHRA Type 2 VHDsand individuals free of VHDs, a CHA2DS2-VASc score was taken to calculated the risk of stroke according to guidelines [1, 14]. Hypertension was defined as systolic blood pressure (SBP) ≥ 140 mmHg, or diastolic blood pressure (DBP) ≥ 90 mmHg, or taking antihypertensive agents. Diabetes mellitus was validated according to a fasting blood glucose ≥ 7 mmol/l, or self-reported diabetes, or using anti-diabetes agents. Individuals who smoked at least one cigarette per day were classified as current smokers, and subjects who drank at least once per week were considered as alcohol drinkers.

### Laboratory measurements

Blood samples were drawn from fasting patients by venipuncture within 24 h after admission, and stored by either ethylene diamine tetra acetic acid (EDTA) or plain tubes according to the clinical laboratory requirements. Serum creatinine and ALT were measured using clinical laboratory methods (Beckman CX9, USA). Glomerular filtration rate (eGFR) was estimated using the Modification of Diet in Renal Disease (MDRD) study equation for Chinese patients [15], and an eGFR < 60 ml/min per 1.73 m^2^ was defined as renal impairment.

### Follow-up and outcomes

Annual clinic visits or telephone interviews were administered by research physicians until death or December 2017. The primary outcome of interest was all-cause mortality. The incidences of heart failure, acute coronary syndrome (ACS), hypotension (SBP < 90 mmHg with symptoms, such as dizziness, fatigue, episodes of syncope, or requiring intravenous infusion of pressors), transient ischemic attack (TIA) or stroke, ventricular tachycardia or fibrillation were also recorded. In patients treated with warfarin, regularly testing international normalized ratio (INR) and prothrombin time (STAGO, France) were recommended during the follow-up period. Time in therapeutic range (TiTR) was determined by the Rosendaal method [16] in patients from whom at least three continuous monitored INR values were available. A TiTR ≥ 66% was taken as good-quality anticoagulation control.

### Statistical analysis

The data were expressed as means and standard deviations (SDs) or median and interquartile range (IQR) for numerical variables and as frequency (n) and percentage (%) for categorical variables. A Kolmogorov–Smirnov test was performed on continuous variables to examine whether there was a normal distribution. Besides, continuous variables were analyzed via one-way analysis of variance or Mann–Whitney U-test as appropriate. Pearson’s Chi-square test or Fisher’s exact test was used for analysis of categorical variables. eGFR was modeled continuously as well as in categories (≥ 90, 60–89, and < 60 ml/min per 1.73 m^2^) according to the Kidney Disease Improving Global Outcomes guidelines [3]. Risk factors for all-cause death were assessed by multivariate Cox regression analysis. A P value < 0.05 was thought as statistically significant (two-tailed test). All statistical analyses were calculated via Statistical Package for Social Sciences software (SPSS 19.0 for Windows, IBM, USA). And the Kaplan–Meier curves were determined by GraphPad Prism (Version 5.01, GraphPad software, USA).

## Results

**Fig. 1:**
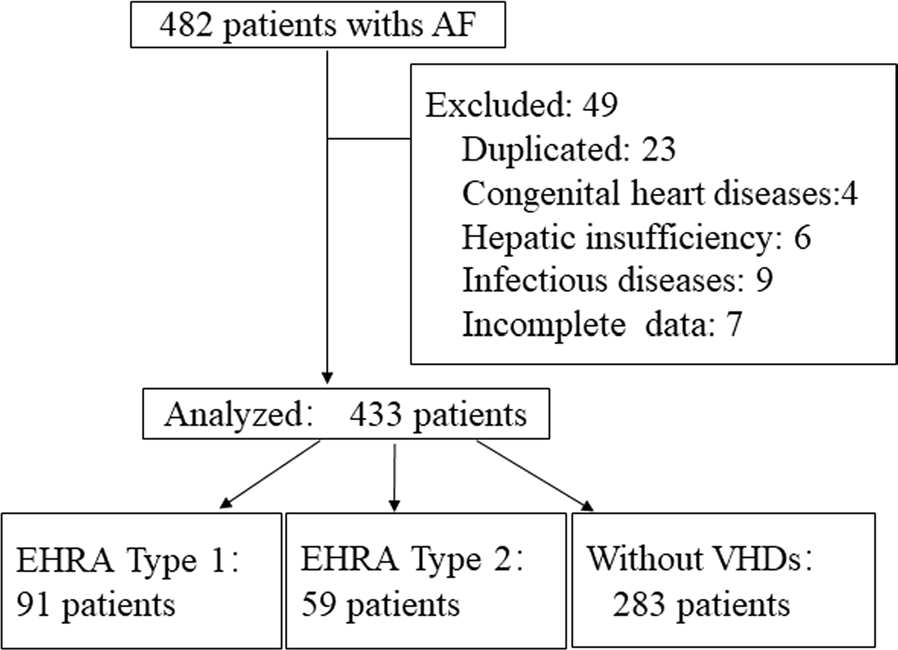
Flowchart of the study. AF indicates atrial fibrillation. *VHD* valvular heart disease

A total of 482 consecutive Chinese patients with AF were screened, and 433 patients with AF entered the final analysis (mean age 65.6 ± 13.2 years, 221(51%) males) (see Fig. 1). Of the study population, more than 80% patients endured persistent or permanent AF, 91 patients (21.0%) were suffering from EHRA Type 1 VHDs, 59 patients (13.6) had EHRA Type 2 VHDs and 283 patients (65.4%) were free of VHDs. The baseline characteristics of study population are presented in Table 1. Mean eGFR of the total population was 70.1 ± 26.9 ml/min per 1.73 m^2^, about 35.6% of them exhibited a renal impairment (eGFR < 60 ml/min per 1.73 m^2^), and no significant difference was observed in renal function among the groups. The proportions of heart failure (> 80%) and permanent AF (> 64%) were higher in patients with VHDs. The mean CHA2DS2-VASc scores were 3.4 ± 1.6 and 3.1 ± 1.8 in patients with EHRA type 2 VHDs and those free of VHDs, respectively. About 27.5% of the total population were treated with oral warfarin and the mean INR value was 1.91 ± 0.77, and 22.7% of them with time in therapeutic range (TiTR) ≥ 66%. There were gradually decreased proportion of taking warfarin, levels of mean INRs, and proportion at adequate TiTR (≥ 66%).

**Table 1:** Baseline characteristics of patients with different types of atrial fibrillation

	Total population n = 433	Individuals with VHDs		Individuals without VHDs n = 283	P value
		ERHA Type 1 n = 91	ERHA Type 2 n = 59		
Male, n (%)	221 (51.0)	29 (31.9)	30 (50.8)	162 (57.2)	< 0.001
Age, years (SD)	65.6 (13.2)	58.0 (11.1)	62.9 (16.0)	65.5 (12.7)	< 0.001
BMI, kg/m^2^(SD)	24.2 (3.8)	22.8 (3.4)	22.8 (3.6)	24.8 (3.7)	< 0.001
SBP, mmHg (SD)	122.8 (22.3)	117.3 (20.3)	123.2 (23.5)	124.5 (22.4)	0.025
Heart rate, beats/min (SD)	102.5 (29.9)	100.5 (29.4)	97.9 (29.0)	104.1 (30.2)	0.273
Duration of AF					< 0.001
Paroxysmal, n (%)	79 (18.2)	11 (12.1)	15 (25.4)	53 (18.7)	
Persistent, n (%)	177 (40.9)	21 (23.1)	6 (10.2)	150 (53.0)	
Permanent, n (%)	177 (40.9)	59 (64.8)	38 (64.4)	80 (28.3)	
Smoking, n (%)	112 (25.9)	12 (13.2)	16 (27.1)	84 (29.7)	0.007
Alcohol, n (%)	36 (8.3)	4 (4.4)	6 (10.2)	26 (9.2)	0.304
Hypertension, n (%)	224 (51.7)	23 (25.3)	31 (52.5)	170 (60.1)	< 0.001
Diabetes, n (%)	171 (39.5)	31 (34.1)	28 (47.5)	112 (39.6)	0.261
Hypercholesterolemia, n (%)	22 (5.1)	0 (0)	7 (11.9)	15 (5.3)	0.005
TIA or Stroke, n (%)	61 (14.1)	11 (12.1)	7 (11.9)	43 (15.2)	0.661
CADs, n (%)	107 (24.7)	5 (5.5)	11 (18.6)	91 (32.2)	< 0.001
Heart failure, n (%)	243 (56.3)	74 (81.3)	51 (86.4)	118 (41.7)	< 0.001
CHA2DS2-VASc Score					
Mean (SD)	–	–	3.4 (1.6)	3.1 (1.8)	–
Median (IQR)	–	–	3 (2–4)	3 (2–4)	–
≥ 2(Male) or ≥ 3(female), n (%)	–	–	49 (83.1)	211 (74.6)	–
ALT, u/l (IQR)	19 (14.0–31.3)	18 (14–27)	23 (15–40)	19 (14–32)	0.223
eGFR, ml/(min·1.73 m^2^)	70.1 (26.9)	73.2 (25.1)	64.9 (27.1)	70.1 (27.4)	0.197
eGFR < 60 ml/(min·1.73 m^2^), n (%)	154 (35.6)	28 (30.8)	28 (47.5)	98 (34.6)	0.309
Warfarin, n (%)	119 (27.5)	55 (60.4)	23 (39.0)	41 (14.5)	< 0.001
INR, mean (SD)^a^	1.91 (0.77)	2.16 (0.76)	1.83 (0.75)	1.51 (0.62)	< 0.001
TiTR ≥ 66%, n (%)^a^	27 (22.7)	21 (38.2)	5 (21.7)	2 (4.9)	< 0.001
Antiplatelet agents, n (%)	214 (49.4)	25 (27.5)	23 (39.0)	166 (58.7)	< 0.001
Beta blockers, n (%)	266 (61.4)	48 (52.7)	38 (64.4)	180 (63.6)	0.159
ACEIs/ARBs, n (%)	146 (33.7)	19 (20.9)	22 (37.3)	105 (37.1)	0.014
CCBs, n (%)	87 (20.1)	6 (6.6)	11 (18.6)	70 (24.7)	0.001

VHD indicates valvular heart diseases

CHA2DS2-VASc score was calculated by congestive heart failure, hypertension, age ≥ 75 years (doubled), diabetes mellitus, prior stroke or TIA (doubled), vascular disease, age 65 to74 years and female sex

*BMI* body mass index, *SBP* systolic blood pressure, *AF* atrial fibrillation, *TIA* transient ischemic attack, *CADs* coronary artery diseases, *IQR* interquartile range, *eGFR* stimated Glomerular filtration rate, *ALT* alanine aminotransferase, *INR* international normalized ratio, *TiTR* time in therapeutic range, *ACEI* angiotensin-converting enzyme inhibitor, *ARB* angiotensin II receptor blocker, *CCB* calcium channel blocker

^a^Only patients treated with warfarin were included

as well as stepwise increased proportions of males, smoking, hypertension, CADs, taking antiplatelet agents and CCBs among patients with EHRA Type 1 VHDs, Type 2 VHDs and individuals without VHDs. Patients with EHRA Type 1 VHDs had the lowest SBP and the proportion of taking ACEIs or ARBs. Their heart rate, the proportions of drinking, diabetes, TIA or stroke and taking β blockers were not significantly different among the groups.

The patients’ clinical outcomes are presented in Table 2. No patient left the study during a median follow-up period of 3.1 (0.5–4.5) years, but 73 patients (16.9%) died. Patients with EHRA Type 2 and free of VHDs had higher incidence of heart failure than those with EHRA Type 1 VHDs (11.9%, 5.7% vs 1.1%, P = 0.019). The incidence of TIA or stroke were 6.6%, 1.7% and 11.3% in AF patients with EHRA Type 1, EHRA Type 2 and free of VHDs, respectively (P = 0.042). There was no difference in the incidence of ACS, hypotension, TIA or stroke and ventricular tachycardia or fibrillation among the groups.

**Table 2:** Clinical outcomes during the follow-up period

Outcomes	Total population n = 433	Individuals with VHDs		Individuals without VHDs n = 283	P-value
		ERHA Type 1 n = 91	ERHA Type 2 n = 59		
Death, n (%)	73 (16.9)	19 (20.9)	10 (16.9)	44 (15.5)	0.497
Heart failure, n (%)	24 (5.5)	1 (1.1)	7 (11.9)	16 (5.7)	0.019
ACS, n (%)	6 (1.4)	1 (1.1)	0 (0)	5 (1.8)	0.375
Hypotension, n (%)	3 (0.7)	0 (0)	0 (0)	3 (1.1)	0.278
TIA or Stroke, n (%)	39 (9.0)	6 (6.6)	1 (1.7)	32 (11.3)	0.042
VT or VF, n (%)	2 (0.5)	1 (1.1)	0 (0)	1 (0.4)	0.395
Others^a^ , n (%)	2 (0.5)	0 (0)	1 (1.7)	1 (0.4)	0.605

Median follow-up period 3.1 (0.5–4.5) years. VHD indicates valvular heart diseases

*ACS* acute coronary syndrome, *TIA* transient ischemic attack, *VT* ventricular tachycardia, *VF* ventricular fibrillation, *AF* atrial fibrillation

^a^Others includes 1 allergic dermatitis and 1 cholecystectomy

**Fig. 2:**
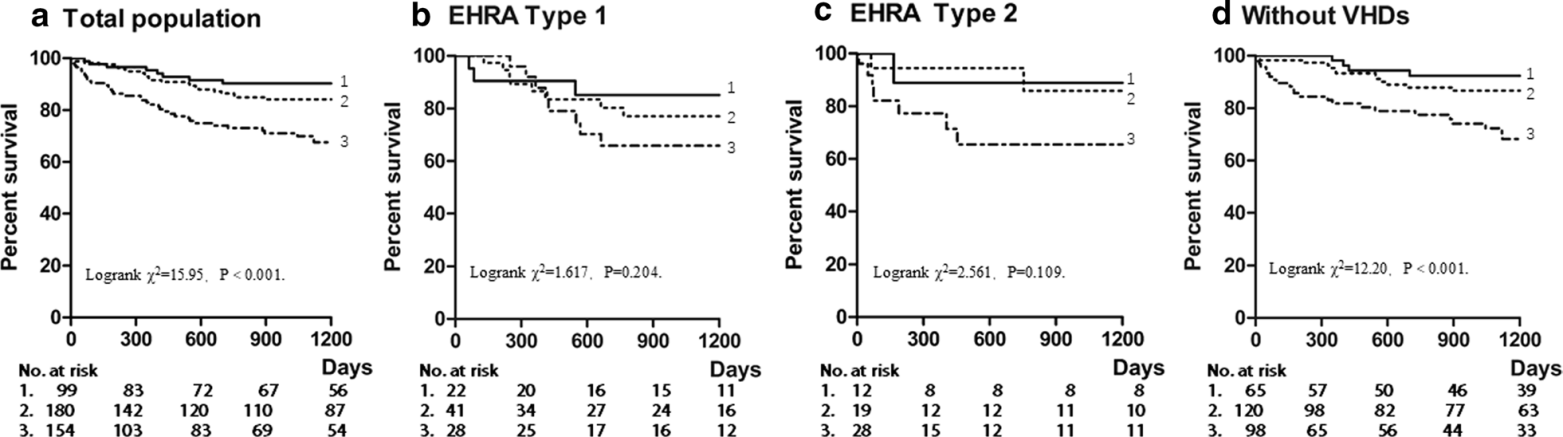
Kaplan–Meier estimates of the incidence of all-cause death. **a** Total population, **b** individuals with EHRA Type 1 VHDs, **c** individuals with EHRA Type 2 VHDs, **d** individuals without VHDs. 1. eGFR ≥ 90 ml/(min·1.73 m^2^), 2. eGFR 60–89 ml/(min·1.73 m^2)^, 3. eGFR < 60 ml/(min·1.73 m^2^). VHD indicates valvular heart disease. *eGFR* estimated Glomerular filtration rate

In univariate Cox regression analysis in which eGFR served as a continues variable (see Table 3), eGFR showed a significant inverse correlation for all-cause death in total population (HR 0.982, 95% CI 0.971–0.993, P = 0.001), and in patients free of VHDs (HR 0.974, 95%CI 0.959–0.990, P = 0.001). Figure 2 shows the Kaplan–Meier curves for all-cause death. Renal impairment (eGFR < 60 ml/min per 1.73 m^2^) significantly increased the risk of all-cause death in both total population (Logrank χ^2^ = 15.95, P < 0.001) and individuals free of VHDs (Logrank χ^2^ = 12.20, P < 0.001), but not in patients with either EHRA Type 1 or Type 2 VHDs (Logrank χ^2^ = 1.617, 2.561.

**Table 3:** Univariate Cox regression analysis of all-cause death in patients with atrial fibrillation

	HR represents	Total population n = 433	EHRA Type 1 n = 91	ERHA Type 2 n = 59	Patients without VHDs n = 283
Male	Versus female	*2.182 (1.332–3.576), 0.002*	1.761 (0.708–4.384), 0.224	1.999 (0.516–7.745), 0.316	*3.086 (1.483–6.421), 0.003*
Age	Per 1-year increment	*1.024 (1.004–1.045), 0.017*	*1.052 (1.007–1.100), 0.024*	1.029 (0.982–1.078),0.229	1.018 (0.991–1.045), 0.189
BMI (kg/m^2^)	Per 1 kg/m^2^ increment	*0.908 (0.848–0.973), 0.006*	*0.812 (0.693–0.952), 0.010*	0.899 (0.730–1.108),0.319	0.957 (0.878–1.043), 0.317
Systolic blood pressure	Per 1 mmHg increment	0.989 (0.978–1.001), 0.073	0.987 (0.964–1.010), 0.274	0.993 (0.965–1.022), 0.653	0.990 (0.975–1.005),0.202
Heart rate	Per 1 bpm increment	*0.990 (0.982–0.998), 0.016*	0.987 (0.970–1.004), 0.139	0.992 (0.969–1.016), 0.513	0.992 (0.982–1.002), 0.110
Duration of AF					
Persistent	Versus Paroxysmal	0.781 (0.365–1.670), 0.524	1.743 (0.181–16.767), 0.630	3.024 (0.424–21.541),0.269	0.650 (0.264–1.597), 0.347
Permanent	Versus Paroxysmal	*2.128 (1.068–4.237), 0.032*	3.409 (0.450–25.818), 0.235	1.348 (0.272–6.688),0.714	*2.492 (1.061–5.851), 0.036*
Smoking	Yes versus no	*1.844 (1.149–2.960), 0.011*	1.136 (0.331–3.898), 0.840	1.655 (0.466–5.878), 0.436	*2.439 (1.336–4.415), 0.004*
Hypertension	Yes versus no	0.729 (0.459–1.158), 0.181	0.994 (0.230–2.092), 0.516	0.995 (0.288–3.440),0.993	0.736 (0.407–1.331), 0.311
Diabetes	Yes versus no	1.200 (0.754–1.910), 0.442	1.368 (0.550–3.402), 0.500	0.411 (0.106–1.591),0.198	1.447 (0.798–2.623), 0.224
Hypercholesterolemia	Yes versus no	1.426 (0.348–5.851), 0.622	NA	1.584 (0.199–12.609),0.664	1.246 (0.169–9.181), 0.829
TIA or stroke	Yes versus no	1.181 (0.621–2.244), 0.612	1.725 (0.502–5.937), 0.387	1.430 (0.179–11.388),0.736	1.072 (0.477–2.410), 0.866
CADs	Yes versus no	1.485 (0.900–2.451), 0.122	0.044 (0.000–91.652), 0.424	0.419 (0.053–3.309),0.409	*2.537 (1.392–4.622), 0.002*
Heart failure	Yes versus no	*3.393 (1.967–5.853), < 0.001*	2.422 (0.559–10.503), 0.237	NA	*3.872 (2.059–7.283), < 0.001*
CHA2DS2-VASc Score	Per 1 score increment	NA	NA	1.000 (0.656–1.525),0.999	1.159 (0.986–1.363), 0.074
ALT	Per 1 U/l increment	*1.002 (1.001–1.004), < 0.001*	1.001 (0.998–1.004), 0.506	0.917 (0.839–1.002), 0.056	*1.003 (1.001–1.004), < 0.001*
eGFR	Per 1 ml/(min·1.73 m^2^) increment	*0.982 (0.971–0.993), 0.001*	0.990 (0.970–1.009), 0.292	0.993 (0.965–1.022), 0.634	*0.974 (0.959–0.990), 0.001*
Warfarin	Yes versus no	1.065 (0.637–1.781), 0.811	0.680 (0.276–1.675),0.402	1.054 (0.297–3.736), 0.935	1.098 (0.464–2.600), 0.832
TiTR	≥ 66% versus < 66%	0.551 (0.173–1.750), 0.312	0.533 (0.155–1.830), 0.317	NA	NA

HR indicates hazard ratio

Data are presented as HR (95% confidential interval), P value

CHA2DS2-VASc score was calculated by congestive heart failure, hypertension, age ≥ 75 years (doubled), diabetes mellitus, prior stroke or TIA (doubled), vascular disease, Age 65 to74 years, female Sex

*NA* not available, *VHD* valvular heart diseases, *BMI* body mass index, *TIA* transient ischemic attack, *CAD* coronary artery disease, *IQR* interquartile range, *GFR* estimated Glomerular filtration rate, *AF* atrial fibrillation

P = 0.204, 0.109, respectively). Table 4 shows the final multivariate Cox proportional models of predictors for all-cause death. After adjustment for multiple relevant co-variables, eGFR was still an independent predictor for all-cause death in total population (HR 0.984, 95% CI 0.972–0.995.

**Table 4:** Multivariable Cox regression analysis of all-cause death in patients with atrial fibrillation, eGFR as a continues variable

Variable	HR represents	HR (95%CI)	P value
Total population			
Age	Per 1-year increment	1.018 (0.999–1.038)	0.066
Male	Versus female	2.418 (1.445–4.046)	0.001
Duration of AF			< 0.001
Persistent	Versus paroxysmal	0.781 (0.352–1.737)	0.545
Permanent	Versus paroxysmal	2.379 (1.156–4.896)	0.019
Warfarin	Yes versus no	1.200 (0.661–2.179)	0.548
TiTR	≥ 66% versus < 66%	0.571 (0.137–1.656)	0.243
eGFR	Per 1 ml/(min·1.73 m^2^) increment	0.984 (0.972–0.995)	0.006
Individuals with EHRA Type 1 VHDs			
Age	Per 1-year increment	1.044 (0.997–1.092)	0.067
Male	Versus female	1.817 (0.687–4.808)	0.229
Duration of AF			0.277
Persistent	Versus paroxysmal	1.994 (0.196–20.257)	0.560
Permanent	Versus paroxysmal	3.937 (0.496–31.278)	0.195
TiTR	≥ 66% versus < 66%	0.491 (0.124–1.945)	0.311
Warfarin	Yes versus no	0.949 (0.334–2.700)	0.922
eGFR	Per 1 ml/(min·1.73 m^2^) increment	0.988 (0.967–1.010)	0.281
Individuals with EHRA Type 2 VHDs			
Age	Per 1-year increment	1.003 (0.976–1.093)	0.269
Male	Versus female	1.439 (0.323–6.413)	0.633
Duration of AF			0.145
Persistent	Versus paroxysmal	33.672 (1.001–1132.496)	0.050
Permanent	Versus paroxysmal	7.210 (0.544–95.538)	0.134
CHA2DS2-VASc score	Per 1 score increment	0.482 (0.214–1.089)	0.079
Warfarin	Yes versus no	1.122 (0.196–6.422)	0.897
eGFR	Per 1 ml/(min·1.73 m^2^) increment	1.000 (0.965–1.037)	0.985
Individuals without VHDs			
Age	Per 1-year increment	0.991 (0.961–1.022)	0.568
Male	Versus female	3.936 (1.780–8.706)	0.001
Duration of AF			0.003
Persistent	Versus paroxysmal	0.561 (0.223–1.412)	0.220
Permanent	Versus paroxysmal	1.866 (0.769–4.528)	0.168
CHA2DS2-VASc score	Per 1 score increment	1.136 (0.927–1.392)	0.217
Warfarin	Yes versus no	1.270 (0.528–3.053)	0.594
eGFR	Per 1 ml/(min·1.73 m^2^) increment	0.975 (0.959–0.992)	0.003

Models were adjusted by age, sex (male vs female), duration of AF, use of warfarin, TiTR (if available), CHA2DS2-VASc score (if available) and eGFR. VHDs indicates valvular heart diseases

CHA2DS2-VASc score was calculated by congestive heart failure, hypertension, age ≥ 75 years (doubled), diabetes mellitus, prior stroke or TIA (doubled), vascular disease, age 65 to74 years, female sex

AF indicates atrial fibrillation

*TiTR* time in therapeutic range, *CI* confidential interval, *HR* hazard ratio, *eGFR* estimated Glomerular filtration rate

P = 0.006) and individuals free of VHDs (HR 0.975, 95% CI 0.959–0.992.

P = 0.003). Additionally, male gender and permanent AF were independent risk factors for all-cause death in total population and individuals free of VHDs.

As shown in Table 5, eGFR served as a category variable, the risk of all-cause death in patients with eGFR < 60 ml/min per 1.73 m^2^ was 1.969 times higher than those with eGFR > 90 ml/min per 1.73 m^2^ in total population. The similar results were exhibited in AF patients free of VHDs, but not in those patients with either EHRA type 1 or type 2 VHDs. In AF patients without VHDs, a receiver operating characteristic (ROC) analysis revealed that area under the curve (AUC) of eGFR for all-cause death [AUC: 0.637 (95% CI 0.539–0.735, P = 0.004)] was higher than that of CHA2DS2-VASc score [AUC:0. 565, (95% CI 0.480–0.649, P = 0.172)]. A combination of eGFR and CHA2DS2-VASc score (AUC = 0.640, 95% CI0.547–0.727, P = 0.004) did not improve the power in predicting the risk of mortality. The best eGFR cutoff point was 60 ml/min per 1.73 m^2^, with a sensitivity of 55.8% and a specificity of 66.5%. While a cutoff point of 30 ml/min per 1.73 m^2^ corresponded to a low sensitivity of 9.3% but a high specificity of 98.6%.

**Table 5:** Multivariable Cox regression analysis of all-cause death in patients with atrial fibrillation, eGFR as a category variable

Variable	HR represents	HR (95%CI)	P value
Total population			
Age	Per 1-year increment	1.018 (0.999–1.038)	0.063
Male	Versus female	2.441 (1.475–4.040)	0.001
Duration of AF			< 0.001
Persistent	Versus paroxysmal	0.747 (0.347–1.608)	0.455
Permanent	Versus paroxysmal	2.187 (1.095–4.370)	0.027
Warfarin	Yes versus no	1.329 (0.753–2.348)	0.326
TiTR	≥ 66% versus < 66%	0.420 (0.122–1.443)	0.168
eGFR			0.001
60–89 ml/(min·1.73 m^2^)	Versus ≥ 90 ml/(min·1.73 m^2^)	1.304 (0.618–2.750)	0.485
< 60 ml/(min·1.73 m^2^)	Versus ≥ 90 ml/(min·1.73 m^2^)	2.969 (1.447–6.093)	0.003
Individuals with EHRA Type 1 VHDs			
Age	Per 1-year increment	1.044 (0.996–1.095)	0.074
Male	Versus female	1.912 (0.692–5.284)	0.212
Duration of AF			0.367
Persistent	Versus paroxysmal	2.006 (0.202–19.970)	0.553
Permanent	Versus paroxysmal	3.525 (0.450–27.638)	0.231
TiTR	≥ 66% versus < 66%	0.452 (0.112–1.823)	0.264
Warfarin	Yes versus no	0.932 (0.326–2.660)	0.895
eGFR			0.339
60–89 ml/(min·1.73 m^2^)	Versus ≥ 90 ml/(min·1.73 m^2^)	1.264 (0.316–5.049)	0.740
< 60 ml/(min·1.73 m^2^)	Versus ≥ 90 ml/(min·1.73 m^2^)	2.522 (0.587–10.843)	0.214
Individuals with EHRA Type 2 VHDs			
Age	Per 1-year increment	1.075 (1.004–1.151)	0.038
Male	Versus female	2.806 (0.640–12.315)	0.171
Duration of AF			0.173
Persistent	Versus paroxysmal	13.838 (0.877–218.340)	0.062
Permanent	Versus paroxysmal	3.615 (0.583–22.423)	0.167
CHA2DS2-VASc score	Per 1 score increment	0.619 (0.328–1.168)	0.138
Warfarin	Yes versus no	2.447 (0.516–11.603)	0.260
eGFR			0.187
60–89 ml/(min·1.73 m^2^)	Versus ≥ 90 ml/(min·1.73 m^2^)	0.517 (0.036–7.409)	0.517
< 60 ml/(min·1.73 m^2^)	Versus ≥ 90 ml/(min·1.73 m^2^)	2.265 (0.217–23.591)	0.494
Individuals without VHDs			
Age	Per 1-year increment	0.993 (0.963–1.023)	0.641
Male	Versus female	3.593 (1.684–7.667)	0.001
Duration of AF			0.003
Persistent	Versus paroxysmal	0.586 (0.234–1.465)	0.253
Permanent	Versus paroxysmal	1.921 (0.784–4.707)	0.153
CHA2DS2-VASc score	Per 1 score increment	1.121 (0.915–1.374)	0.271
Warfarin	Yes versus no	1.307 (0.542–3.151)	0.550
eGFR			0.007
60–89 ml/(min·1.73 m^2^)	Versus ≥ 90 ml/(min·1.73 m^2^)	1.433 (0.546–3.763)	0.465
< 60 ml/(min·1.73 m^2^)	Versus ≥ 90 ml/(min·1.73 m^2^)	3.660 (1.402–9.556)	0.008

Models were adjusted by age, sex (male vs female), duration of AF, use of warfarin, TiTR (if available), CHADS-VASc score (if available) and eGFR. VHDs indicates valvular heart diseases

CHA2DS2-VASc score was calculated by Congestive heart failure, Hypertension, Age ≥ 75 years (doubled), Diabetes mellitus, Prior Stroke or TIA (doubled), Vascular disease, Age 65 to74 years, female Sex. AF indicates atrial fibrillation

*TiTR* time in therapeutic range, *CI* confidential interval, *HR* hazard ratio, *eGFR* estimated Glomerular filtration rate

## Discussion

Renal impairment is a common comorbidity in patients with AF. In present study, we demonstrated that reduced eGFR was a poor prognostic factor of long-term clinical outcomes among Chinese patients with AF, especially among those free of VHDs. Therefore, dynamic monitoring and protecting against progressive deterioration of renal function might help to improve the prognosis of patients with AF.

AF is associated with doubled risk of all-cause mortality [1, 2]. The CHADS2 and CHA2DS2-VASc score are common algorithms applied for tailoring stroke risk in AF patients with EHRA type 1 VHDs and free of VHDs [1, 14], and these algorithms have simplified the initial decision for oral anticoagulants in such patients [6, 9]. Previous studies had explored clinical biomarkers for risk stratification in selected patients with AF and provided improved decision on assessing the risks of stroke or systemic embolism and bleeding [17, 18]. eGFR, a substituted indicator of renal function, could be calculated according to common clinical data (age, sex and serum creatine) [15], but did not increase economic burden of individual patients. There were increasing studies to evaluate the relationship between eGFR and clinical outcomes in different clinical settings. Previous studies had shown that reduced eGFR increased the risk of adverse outcomes in patients with non-valvular AF [12] at either low or high risk of stroke [6, 19]. In a large community-based study, an independent, graded association was observed between renal impairment (eGFR ≤ 60 mL/min per 1.73 m^2^) and the risk of death, cardiovascular events, and hospitalization [20]. In addition, reduced eGFR during the follow-up period was associated with AF recurrence among patients after AF catheter ablation [9]. Our study also found that a reduced eGFR was associated with higher risk for mortality among Chinese patients with AF, especially in the subgroup patients free of VHDs.

CKD is a prevalent health problem associated with cardiovascular mortality [4, 5]. Our study indicated that reduced eGFR (eGFR < 60 mL/min per 1.73 m^2^) was about one third in Chinese patients with AF. Moreover, even slightly reduced eGFR might increase the risk of major adverse events associated with death [19]. In our subgroup analysis, reduced eGFR was associated with higher mortality in Chinese patients free of VHDs but not with VHDs. To our knowledge, rheumatic diseases are the common VHDs leading to AF, and the AF patients with VHDs are relatively younger and have fewer comorbidities. The prevalence of AF in patients free of VHDs increased with age, smoking, higher BMI, hypertension, diabetes, hypercholesterolemia and CADs, which are known risk factors for renal impairment and poor clinical outcomes. Thus, it was required to collect medical history carefully, understand comprehensively and manage accompanied clinical settings, such as smoking, hypertension, diabetes mellitus, hypercholesterolemia and CADs effectively.

There are several inherent limitations to the current study. First, the main limitation of this study is that it represents a retrospective study with limited sample. Second, only eGFR has been evaluated in this study, other indicators associated with renal function, such as albuminuria, cystatin C and GDF-15, were not obtained. Third, guideline- recommended anticoagulation was still insufficient. Non-vitamin K antagonist oral anticoagulants (NOACs) are recommended in preference to vitamin K antagonists for stroke prevention in high risk AF patients without EHRA type I VHDs [1]. And taking NOACs was conducive to postponing the deterioration of renal function compared to use of warfarin [21]. However, NOACs were not always available due to their high costs and unsupported by local health insurance in China, thus warfarin was still the only recorded oral anticoagulant in our study population. There were still inadequate in prescribing oral anticoagulants and getting an ideal TiTR, especially in AF patients free of VHDs, although they were at high risk of stroke. Also, we have just taken all-cause death as the primary endpoint, however, other adverse outcomes, such as heart failure, myocardial infarction and stroke were ignored due to limited information. Thus, despite adjustment of a series of confounding variables, there were some unrecognized factors which might affect the judgement of study results. Therefore, a large sample prospective study is still needed to evaluate the relationship between renal function and clinical outcomes.

## Conclusions

In summary, renal impairment is a common clinical setting in Chinese patients with AF, and a reduced eGFR is independently associated with a worse prognosis among Chinese patients with AF, especially in those patients free of VHDs. Closely monitoring renal function, guideline-recommended anticoagulation and actively preventing renal impairment might improve the prognosis of these patients.

## Data Availability

The datasets used in this study are available from the corresponding author on reasonable request.

## Abbreviations

ACEI: Angiotensin-converting enzyme inhibitor
ACS: Acute coronary syndrome
AF: Atrial fibrillation
AHA: American Heart Association
ALT: Alanine aminotransferase
ARB: Angiotensin II receptor blocker
AUC: Area under curve
BMI: Body mass index
CAD: Coronary artery disease
CHA2DS2-VASc score: Calculated according to congestive heart failure, hypertension, age ≥75 years (doubled), diabetes mellitus, prior stroke or TIA (doubled), vascular disease, age 65 to74 years and female sex
CCB: Calcium channel blocker
CI: Confidence interval
CKD: Chronic kidney disease
DBP: Diastolic blood pressure
eGFR: Estimated glomerular filtration rate
GDF: Growth differentiation factor
HR: Hazard ratio
INR: International normalized ratio
IQR: Interquartile range
MDRD: Modification of diet in renal disease
ROC: Receiver operating characteristic curve
SBP: Systolic blood pressure
SD: Standard deviation
TIA: Transient ischemic attack
TiTR: Time in therapeutic range
VHD: Valvular heart disease
